# Most Important Factors for the Implementation of Shared Decision Making in Sciatica Care: Ranking among Professionals and Patients

**DOI:** 10.1371/journal.pone.0094176

**Published:** 2014-04-07

**Authors:** Stefanie N. Hofstede, Leti van Bodegom-Vos, Manon M. Wentink, Carmen L. A. Vleggeert-Lankamp, Thea P. M. Vliet Vlieland, Perla J. Marang-van de Mheen, for the DISC study group

**Affiliations:** 1 Department of Medical Decision Making, Leiden University Medical Center, Leiden, The Netherlands; 2 Department of Neurosurgery, Leiden University Medical Center, Leiden, The Netherlands; 3 Department of Orthopaedics, Leiden University Medical Center, Leiden, The Netherlands; Northwestern University, United States of America

## Abstract

**Introduction:**

Due to the increasing specialization of medical professionals, patients are treated by multiple disciplines. To ensure that delivered care is patient-centered, it is crucial that professionals and the patient together decide on treatment (shared decision making (SDM)). However, it is not known how SDM should be integrated in multidisciplinary practice. This study determines the most important factors for SDM implementation in sciatica care, as it is known that a prior inventory of factors is crucial to develop a successful implementation strategy.

**Methods:**

246 professionals (general practitioners, physical therapists, neurologists, neurosurgeons, orthopedic surgeons) (30% response) and 155 patients (96% response) responded to an internet-based survey. Respondents ranked barriers and facilitators identified in previous interviews, on their importance using Maximum Difference Scaling. Feeding back the personal top 5 most important factors, each respondent indicated whether these factors were barriers or facilitators. Hierarchical Bayes estimation was used to estimate the relative importance (RI) of each factor.

**Results:**

Professionals assigned the highest importance to: quality of professional-patient relationship (RI 4.87; CI 4.75–4.99); importance of quick recovery of patient (RI 4.83; CI 4.69–4.97); and knowledge about treatment options (RI 6.64; CI 4.53–4.74), which were reported as barrier and facilitator. Professionals working in primary care had a different ranking than those working in hospital care. Patients assigned the highest importance to: correct diagnosis by professionals (barrier, RI 8.19; CI 7.99–8.38); information provision about treatment options and potential harm and benefits (RI 7.87; CI 7.65–8.08); and explanation of the professional about the care trajectory (RI 7.16; CI 6.94–7.38), which were reported as barrier and facilitator.

**Conclusions:**

Knowledge, information provision and a good relationship are the most important conditions for SDM perceived by both patients and professionals. These conditions are not restricted to one specific disease or health care system, because they are mostly professional or patient dependent and require healthcare professional training.

## Introduction

Sciatica is a common disorder with prevalence reported up to 43% [1]. It is mostly caused by a herniated disc with compression of the nerve root, which gives radiating leg pain. Seventy percent of patients with sciatica recover in the first 6–8 weeks with conservative treatment [2]. After 6–8 weeks it is possible to consider prolonged conservative treatment or surgery. Care to sciatica patients is given by various disciplines: the general practitioner, physical therapist, neurologist and neurosurgeon or orthopedic surgeon are frequently involved.

A large, randomized clinical trial showed no significant difference in clinical outcomes between conservative treatment and surgery after 1 and 2 years in patients with sciatica [3]. Other, low quality studies showed conflicting results [4]. As the literature is not consistent regarding the best treatment option [3], [4], the choice can be considered preference sensitive. Therefore, the Dutch multidisciplinary sciatica guideline [5] recommends to integrate shared decision making (SDM) in consultations. In SDM, clinicians and the patient make decisions jointly, weighting the best available evidence regarding different treatment options [6]. Patients are encouraged to consider prolonged conservative treatment or surgery and the likely benefits and harm of each so that they communicate their preferences and help to select the best treatment for them. Only when professionals and the patient together decide on treatment (SDM), delivered care can be truly patient-centered.

Despite the recommendation to integrate SDM in consultations [5], there are strong indications that SDM is not yet adopted in clinical management of patients with sciatica. Recently, a comparison between regions in the Netherlands showed considerable variation in the number of sciatica patients that undergo surgery, ranging from 31 to 140 per 100,000 inhabitants [7]. In addition, Dutch surgery rates for sciatica patients are four times higher than in the United Kingdom and two times higher than in Sweden [7] while The United States have a 40% higher surgery rate than the Netherlands [8]. As enhancing the use of SDM was found to be associated with lower hospital admission rates through the prevention preference-sensitive surgeries [9], its use is likely to play a role in the variation in surgery, in addition to factors such as case mix.

Previous research concerning the barriers for implementation of SDM in clinical practice mainly focussed on one discipline (monodisciplinary) or on inter-professional (IP) teams [10]–[12]. An inter-professional approach involves separate disciplines that integrate different approaches mostly into a single consultation [13]. However, due to the increasing specialization of medical professionals, patients nowadays are treated by multiple disciplines in several separate consultations as for example in sciatica care were the general practitioner, physical therapist, neurologist and neurosurgeon or orthopedic surgeon are frequently involved. SDM in multidisciplinary care utilizes the skills and experience of professionals from different disciplines, with each discipline approaching the patient from their own perspective [13], so that different barriers and facilitators for SDM implementation may play a role and to a different extent than in a monodisciplinary setting or in an inter-professional team. This is currently unknown. Furthermore, most studies focus on professionals only, while patients are part of the SDM process and may perceive other barriers and facilitators which may be also important for the implementation of SDM. To our knowledge, this is the first quantitative study that focuses on barriers and facilitators of SDM perceived by professionals of different disciplines as well as patients.

In a previous qualitative study among patients and professionals we explored the full spectrum of barriers and facilitators related to the use of SDM in sciatica care, including those related to the multidisciplinary setting [14]. However, these qualitative data do not provide the importance of these barriers and facilitators for SDM implementation. This is needed to focus an implementation strategy towards the most important barriers and facilitators. Therefore this study aims to answer the following research questions:

1. Which factors are most important for SDM implementation in multidisciplinary sciatica care?
2. Are these factors mainly a barrier or a facilitator for SDM?

## Methods

### Setting

In the Netherlands, the diagnosis sciatica is mostly made by general practitioners (GPs). The Dutch multidisciplinary guideline recommends conservative treatment during the first 6–8 weeks, provided when severe neurologic symptoms are lacking. After 6–8 weeks patients are usually referred to a neurologist for further investigation if symptoms continue. The neurologist evaluates the presence of a radicular pain syndrome and orders an MRI to visualize the affected spinal nerve(s) and to judge possible compression. If the MRI confirms a nerve compressing herniated disc, a surgical intervention can be considered, but it is also possible to choose prolonged conservative treatment. In case of surgery, the neurologist will refer the patient to a neurosurgeon or orthopedic surgeon for further surgical decision making.

### Population

We randomly selected 200 general practitioners (GPs), 200 physical therapists (PTs), 200 neurologists and 200 orthopedic surgeons from the Dutch medical address book, which includes most professionals in The Netherlands. All Dutch neurosurgeons (n = 131) were invited to participate in the study. Patients were recruited via advertisements in local newspapers across the Netherlands. In addition, the professionals interviewed in our previous study were asked to recruit patients. We aimed to include at least 100 patients. We included sciatica patients diagnosed in the last 12 months, 18 years and older and able to understand written Dutch instructions. Questionnaires were sent in November 2012. Non-responders (professionals and patients) received two reminders, each within a period of 1.5 weeks. Participants who completed the questionnaire received a ten euro gift card as an incentive.

### Survey development and deployment

We developed two different internet-based surveys, one for professionals and one for patients, as the barriers and facilitators identified in the previous qualitative study differed between these groups [14]. Each questionnaire consisted of two parts. In the first part we assessed professionals' and patients' preferences for decision making using the control preference scale (CPS) [15]. We asked professionals about their use of shared decision making in routine practice (self-reported), and which discipline should have the leading role in SDM in practice. Furthermore, we asked patients about their care trajectory and the decision making preferences and practice.

For the second part, barriers and facilitators identified in our previous qualitative study were translated into neutral statements. The questionnaire included 53 factors for professionals and 35 factors for patients, that were used in a best-worst scaling (Maximum Difference scaling (MaxDiff)) exercise following an orthogonal design [16]. MaxDiff is an efficient method to rank multiple items. It is easy to complete for respondents, because they only have to choose the most and least important factor within a set. The other factors are then known to be in between those factors. This is more efficient than using Paired Comparisons [17]. Furthermore, the MaxDiff is scale free, and therefore prevents scale-use bias [18]. In this study, respondents were presented with 6 factors at a time. This was repeated a number of times so that all factors were presented in different combinations. To avoid higher importance given to the first mentioned items, the order of items was randomized between respondents. Each item was presented twice [19], and we created 300 versions of the questionnaires to ensure variation in combination of items. At the end of the MaxDiff exercise, each respondent saw their own top five factors, considered as most important given their previous answers. Respondents were asked to indicate for each factor if they perceived it as a barrier or facilitator in their current situation (e.g., knowledge about treatment options can be perceived as a barrier if there is a lack of knowledge, and a facilitator if they have sufficient knowledge). We used Sawtooth Software's SSI Web 8.1 to construct the survey and the MaxDiff exercise.

Finally, we asked the following demographic information of all respondents: age, gender, region (north, middle, and south) and ethnicity. In addition we asked professionals in which setting they work (general hospital, university medical center, private clinic, teaching hospital), and patients educational level. We distinguished three educational level groups: basic education (no or only primary education), intermediate education (prevocational secondary education, senior secondary vocational training, senior secondary general education, preuniversity education), or high education (higher professional education or university (bachelor, master, or PhD degree)).

### Analysis

Descriptive statistics were used for the general characteristics of the respondents. We compared the characteristics (age, gender, ethnicity, discipline and setting), and decision making style (preferences and behavior) of professionals who did and did not complete the questionnaire during the MaxDiff exercise. In addition we examined differences between professionals and patients regarding preferences and perceived practice of SDM use. For these comparisons we used independent T-test, Mann Whitney U, Fisher's exact or *χ* ^2^ tests, as appropriate. Hierarchical Bayes (HB) estimation was used to estimate relative importance scores (RI) for each factor for each respondent, based on the choices made by respondents in the MaxDiff exercise [20]. These scores can be derived even though respondents evaluate only a part of all possible combinations of items [16]. HB estimation uses an iterative process, along with information from other respondents, to estimate the utilities that best fit the choices of each subject. The sum of all RIs is 100 for each individual. Factors more often chosen as most important get a higher RI, whereas factors chosen as least important get a lower RI. Therefore, a high RI indicates that a factor is very important for this individual, whereas a low RI indicates that a factor is less important. To assess which factors on average are the most important factors for the use of SDM in clinical practice, we calculated the RI for each factor over all respondents with its 95% confidence interval. We checked for random responders using the root likelihood (RLH), excluding respondents with a root likelihood less than 208 [21]. The overall RLH was used as a measure of the goodness of fit. We examined differences in RI between primary care and hospital care professionals, as well as differences in decision making using *χ* ^2^ tests. We divided professionals in three groups: professionals who let the patient decide, professionals who make a shared decision, and professionals who decide themselves. Sawtooth Software 8.1 and SPSS 20.0 were used for analyses. Significance testing was done two-sided at α = 0.05.

### Ethical approval

This study protocol (P12.016) was presented to the Medical Ethical Committee of the Leiden University Medical Center. An exemption was obtained, as ethical approval for this type of study is not required under Dutch law.

## Results

### Response

**Figure 1:**
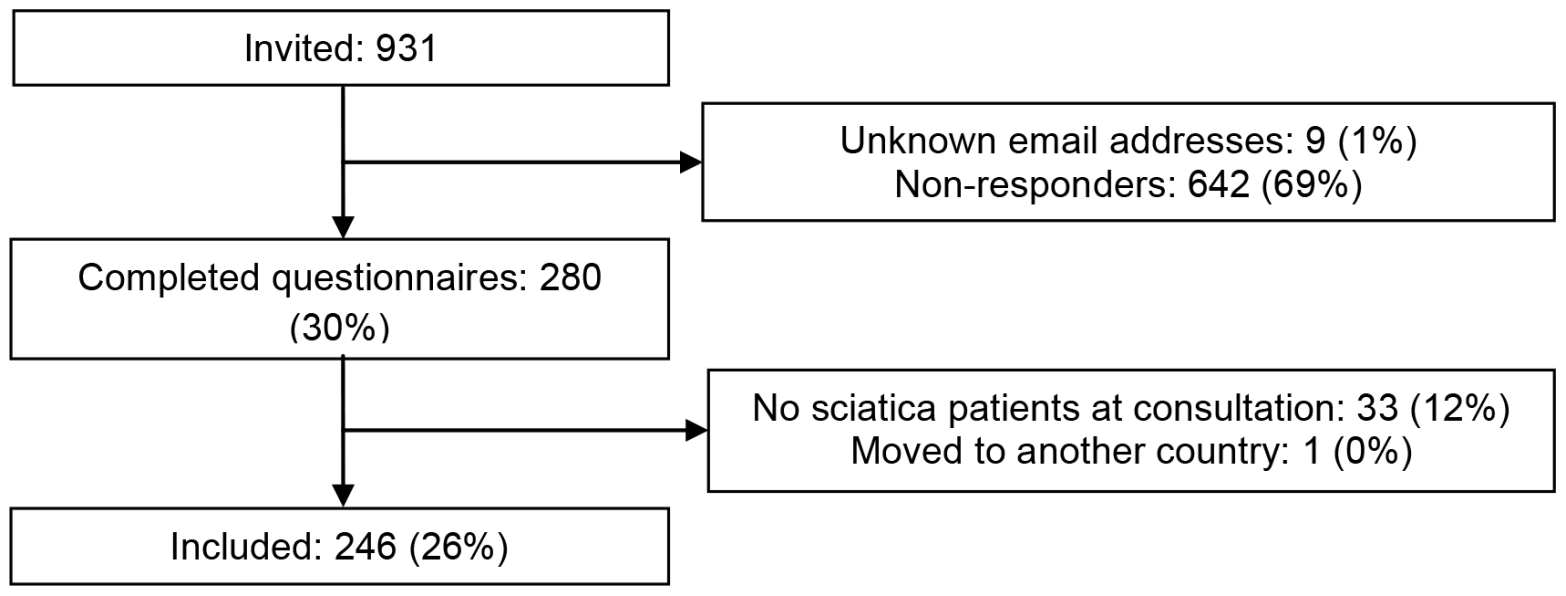
Inclusion and response of professionals.

Figure 1 shows the inclusion and response of professionals after two reminders. A total of 246 professionals completed the questionnaire and were included. A total of 162 patients were invited for participation (91% via advertisement and 9% via professionals). One patient was excluded because he did not have sciatica. 155 patients (96%) completed the questionnaire.

### Characteristics of respondents

Among professionals GP's had the lowest response rate (15%) and neurosurgeons had the highest response rate (36%). Characteristics of professionals and patients who completed the questionnaire are described in table 1. Most professionals were male, and of Dutch origin. Responding and non-responding professionals did not differ in age, gender, ethnicity, discipline and setting (data not shown). Concerning the work area of professionals and residence of patients, respondents came from all regions in the Netherlands (table 1). The majority of patients had an intermediate level of education (table 1).

**Table 1:** Characteristics of participating professionals and patients.

Characteristics	Professionals (n = 246)	Patients (n = 155)
Age, years (mean, SD)	46 (10.0)	50 (13.2)
Sex, no. (%)		
Male	173 (70)	68 (44)
Education, no. (%)		
Basic	-	2 (1)
Intermediate	-	95 (61)
High	-	58 (37)
Ethnicity, no. (%)		
Dutch	198 (80)	149 (96)
Western (except Dutch)	37 (15)	6 (4)
Non-Western	11 (4)	0 (0)
Region, no. (%)	*Work area^a^*	
North	80 (33)	66 (43)
Middle	112 (46)	53 (34)
South	63 (26)	36 (23)
Discipline, no. (%)		
Physical therapist	63 (26)	-
General practitioner	29 (12)	-
Neurologist	58 (24)	-
Neurosurgeon	47 (19)	-
Orthopedic surgeon	49 (20)	-
Setting* (hospital care n = 154), no. (%)		
General hospital	78 (51)	-
University medical center	39 (25)	-
Private clinic	9 (6)	-
Teaching hospital	61 (40)	-

* Multiple options possible

### Current care and SDM

For 118 (76%) patients it was the first time they were diagnosed with sciatica. Of all the patients 120 (77%) had been referred to hospital care, 53 patients (34%) already had surgery, and 5 patients (3%) were on a waiting list for surgery. Visited disciplines were the PT (79%), GP (88%), neurologist (76%), neurosurgeon (47%), the orthopedic surgeon (12%) and others (20%; e.g., anesthesiologist (4%), other therapists (Caesar or mensendieck) (3%), or chiropractor (2%)).

**Figure 2:**
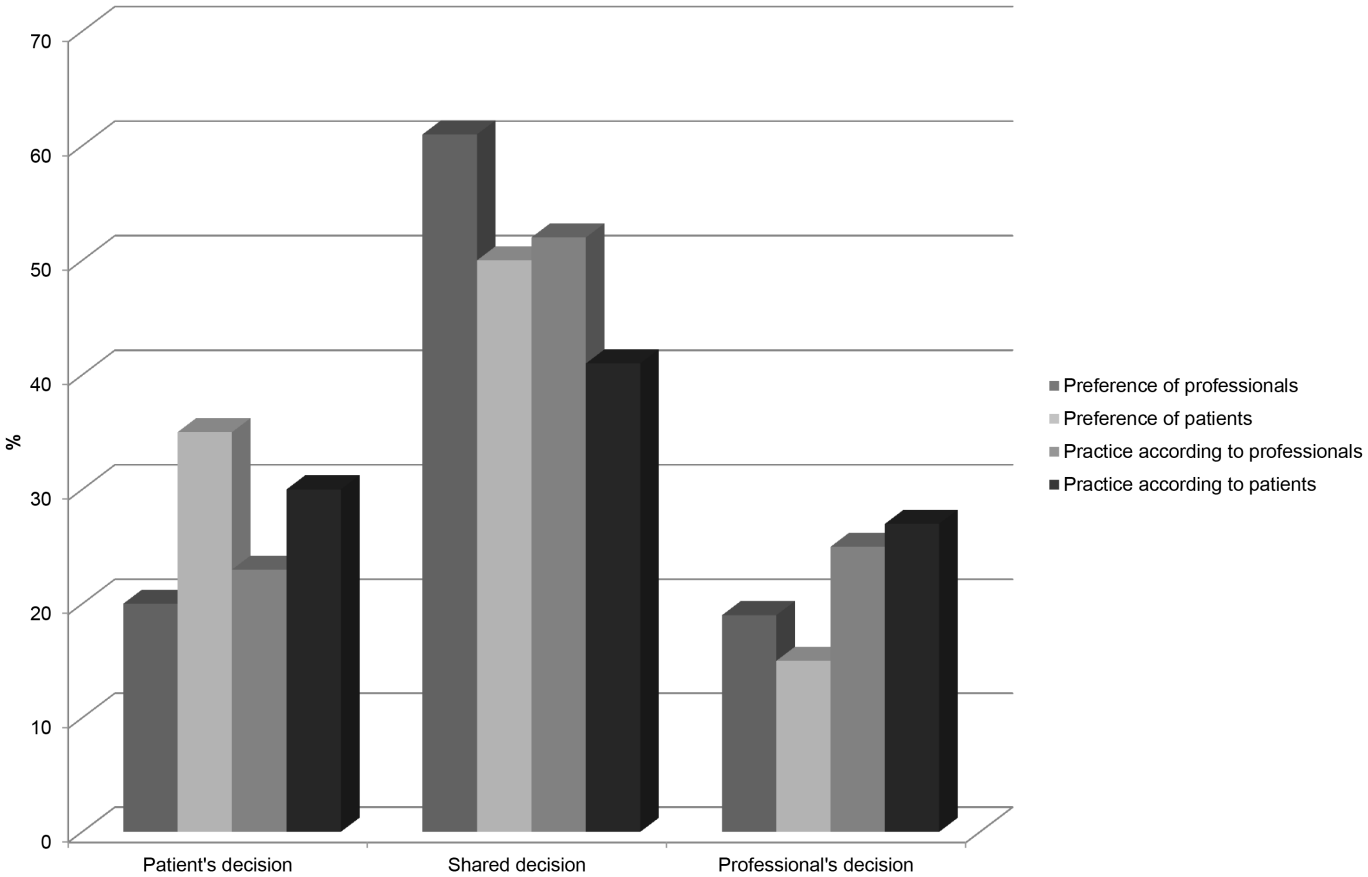
Preferences and practice of decision making in sciatica care according to professionals and patients. Patient's decision: Patient makes the final selection about treatment. Shared decision: the doctor and patient share responsibility for deciding which treatment is best for the patient. Professional's decision: the doctor makes the final decision about treatment.

Figure 2 shows the preferences and practices of decision making in sciatica care according to professionals and patients. The majority of the professionals (61%) said that they prefer a shared decision, whereas 52% stated they actually use SDM in daily practice. Preferences of professionals for SDM and the actual use of SDM in their practice are associated (p<0.001). Fifty percent of the patients said they wanted the decision to be a shared decision. However, only 41% of the patients said they actually made the decision together with the professional in their own situation. These discrepancies between preference and actual use may be explained by different barriers and facilitators.

### Barriers and facilitators for professionals

Table 2 presents the top 10 factors influencing SDM according to professionals. The factors in the tables are the factors presented to participants during the maximum difference exercise, translated from Dutch literally. Most important factors for professionals on average were: quality of professional-patient relationship, importance for quick recovery of patient, and knowledge about treatment options. The higher average RI of these factors means that more participants had this factor in their top 5. However, if there is a lot of variation between participants, for example if part of the respondents rank a factor as most important and another part as least important, the average RI will be lower even though a considerable amount of professionals mentioned this factor in their top 5. For example the factor “ability of patients to make the decision about treatment” has an RI of 4.48 (CI 4.33–4.63), despite the fact that 46% of the professionals mentioned this factor in their top 5, which is higher than the 38% for “knowledge about treatment options” with a slightly higher average RI (4.64 (CI 4.53–4.74) table 2).

**Table 2:** Most important factors for SDM in sciatica care according to professionals.

Factor	Relative importance score (mean, 95% CI)	% professional who reported factor in top 5	% of all top 5 facilitators (n = 1080)	% of all top 5 barriers (n = 150)
1. Quality of professional-patient relationship	4.87 (4.75–4.99)	54	11	11
2. Importance for quick recovery of patient	4.83 (4.69–4.97)	52	11	8
3. Knowledge about treatment options	4.64 (4.53–4.74)	38	7	10
4. Skills to apply SDM	4.53 (4.42–4.65)	36	7	6
5. Ability of patients to make the decision about treatment	4.48 (4.33–4.63)	46	10	7
6. Patients' willingness to decide	4.46 (4.32–4.61)	42	8	11
7. Availability of scientific literature	4.25 (4.09–4.41)	36	8	5
8. Criteria for referral and/or surgery	4.20 (4.05–4.35)	28	6	5
9. Interpretation of information by patients	3.92 (3.76–4.08)	22	4	7
10. Visibility into what other disciplines can do	3.77 (3.62–3.92)	13	2	4

Furthermore, table 2 shows that many factors are both barriers and facilitators, and that factors with the highest RI are also those most frequently mentioned as barriers and facilitators. For example 54% of the professionals reported “quality of professional-patient relationship” in their top 5. This factor represented 11% of all facilitators, and 11% of all barriers in the top 5′s. Overall in the top 5, more factors were perceived as facilitators than as barriers.

We compared the ranking of factors for professionals working in primary (PT and GP) and hospital care (NL, NS and OS). Table 3 shows that the importance of factors from the overall top 10 depends on the work setting. For instance, professionals working in primary care considered “quick recovery of the patient” as more important compared to hospital care (RI 5.05 vs. 4.61) whereas professionals in hospital care found “skills to apply SDM” as more important (RI 4.73 vs. 4.28). Furthermore, professionals in primary care significantly more often considered “clarity of policy for PT's in sciatica” (RI 3.97 vs. 0.82) and “communication between medical disciplines and paramedics” (RI 3.93 vs. 1.47) as important factors for SDM, which both are not in the overall top 10 of most important factors. Their top 10 did not contain the factors “availability of scientific literature” (RI 3.90), and “interpretation of information by patients” (RI 3.21). Professionals in hospital care on the other hand, significantly more often considered “the need for SDM” (RI 4.31 vs. 2.57) as an important factor. Their top 10 also included the “the clarity of the concept SDM” (RI 4.10 vs. 3.19) but did not contain “criteria for referral and/or surgery” (RI 3.64), and “visibility into what other disciplines can do” (RI 3.34).

**Table 3:** Most important factors for SDM in sciatica care, by work setting of professionals and decision making.

Factors in general top 10	Professional who reported factor in top 5 (%)							
		Work setting			Practices in decision making			
	Overall (%)	Primary care (%) (n = 92)	Hospital care (%) (n = 154)	P-value	Patient decide (%) (n = 56)	SDM (%) (n = 128)	Professional decide (%) (n = 62)	P-value
1. Quality of professional-patient relationship	54	46	60	0.032	55	54	55	0.981
2. Importance for quick recovery of patient	52	68	42	<0.001	54	47	61	0.170
3. Knowledge about treatment options	38	37	39	0.754	34	38	44	0.546
4. Skills to apply SDM	36	24	43	0.003	39	38	27	0.282
5. Ability of patients to make the decision about treatment	46	35	53	0.005	57	47	35	0.061
6. Patients' willingness to decide	42	34	47	0.035	48	45	31	0.094
7. Availability of scientific literature	36	36	36	0.938	32	36	40	0.651
8. Criteria for referral and/or surgery	28	48	16	<0.001	29	25	34	0.441
9. Interpretation of information by patients	22	13	27	0.012	29	16	26	0.116
10. Visibility into what other disciplines can do	13	20	9	0.018	13	14	11	0.861

In addition, we examined differences in decision making (self-reported) (table 3). Even though the differences were not statistically significant, it seemed that professionals who would let the patient decide, more often had the “ability of patients to make the decision about treatment” in the top 5 compared to professionals who decide themselves (p = 0.06, table 3). Furthermore, professionals who used SDM in their practice reported “clarity of the concept SDM” (RI 4.19) and “need for SDM” (RI 4.11) as important, whereas their top 10 did not include “interpretation of information by patients” (RI 3.74), and “visibility into what other disciplines can do” (RI 3.89). The top 10 of professionals who make the decision themselves, did not include “visibility into what other disciplines can do” (RI 3.21) but instead “knowledge about the sciatica guideline” (RI 3.97).

### Barriers and facilitators for patients

Table 4 presents the top 10 factors influencing SDM according to patients. Patients on average perceived “correct diagnosis by the professional”, “information provision about treatment options and potential harm and benefits”, and “explanation of the professional about the care trajectory” as the most important factors, given the average RI. However, some factors may be perceived as important by a small group of patients, and thus will have a lower RI on average, which does not necessarily have to mean that these are not important barriers and facilitators. For example “contradictory information of the professionals”, “waiting list for surgery” and “waiting list for a visit to the neurologist” on average have a low importance (RI 1.16 (CI 1.45–1.77), RI 2.36 (CI 1.96–2.76) and RI 2.02 (CI 1.66–2.38), respectively) but relatively many of these patients perceived it as barriers and represented respectively 8%, 8% and 12% of all barriers. So these may be barriers for a smaller group of patients. As for professionals, more factors in the top 5 for patients were perceived as facilitators than as barriers.

**Table 4:** Most important factors for SDM in sciatica care according to patients.

Factor	Relative importance score (mean, 95% CI)	% patients who reported factor in top 5	% of all top 5 facilitators (n = 671)	% of all top 5 barriers (n = 104)
1. Correct diagnosis by professional	8.19 (7.99–8.38)	62	13	3
2. Information provision about treatment options and potential harm and benefits	7.87 (7.65–8.08)	53	10	12
3. Explanation of the professional about the care trajectory	7.16 (6.94–7.38)	37	7	10
4. Confidence in the professional	7.02 (6.82–7.23)	37	8	2
5. Knowledge of the professional	6.94 (6.68–7.20)	38	8	2
6. Guidance in conservative treatment by the professional	6.35 (6.09–6.61)	32	7	4
7. Explanation about the diagnosis sciatica by the professional	6.33 (6.05–6.62)	34	8	0
8. Attention for patient's personal situation	4.98 (4.54–5.43)	31	7	3
9. Attention for patient's preferences	4.71 (4.46–4.96)	17	3	5
10. Information materials about the diagnosis and treatment options and potential harms and benefits	4.24 (3.81–4.67)	17	3	3

## Discussion

This study shows which factors are most important for the implementation of SDM in sciatica care. Overall, more facilitators than barriers were perceived. For professionals the most important factors are “quality of professional-patient relationship”, “importance for quick recovery of patient”, and “knowledge about treatment options”. Patients perceived “correct diagnosis by professional”, “information provision about treatment options and potential harm and benefits”, and “explanation of the professional about the care trajectory” as the most important factors. In short: knowledge, information provision and a good relationship are perceived as important conditions for SDM by both patients and professionals.

Previous research concerning SDM implementation mainly focussed on one discipline (monodisciplinary). Main barriers mentioned in literature included time constraints, the lack of applicability due to patient characteristics or the clinical situation [10]. Main facilitators pertained to the motivation of health professionals, the perception that SDM leads to improved patient outcomes and to improved health care processes [10]. The lack of applicability due to patient characteristics as mentioned in the literature overlaps with some barriers mentioned in professionals top 10 in the current study (e.g., ability of patients to make the decision about treatment), but the other barriers and facilitators reported in the literature are not among the most important barriers and facilitators as reported in the present study. This may be due to the fact that available studies mainly assessed barriers and facilitators to implement SDM in a monodisciplinary setting, whereas sciatica care involves multiple disciplines. Barriers reported in a study related to interprofessional SDM were imbalance of power between health professionals of different disciplines, the existence of professional silos, and disagreement about roles and responsibilities between different disciplines. Main facilitators were mutual knowledge and understanding of disciplinary roles, trust and respect between different disciplines [12]. Visibility into what other disciplines can do and criteria for referral and/or surgery are related to the barrier “disagreement about roles and responsibilities between different disciplines” and the facilitator “understanding of disciplinary roles”, but the other barriers and facilitators reported in this interprofessional SDM study are not among the most important barriers and facilitators as reported in the present study. Furthermore, many studies used qualitative methods [10], [11] allowing an analysis of which barriers and facilitators play a role, but do not provide information on the importance of each barrier or facilitator. The barriers and facilitators most mentioned in our previous qualitative study [14], using interviews and focus groups were not always consistent with the highest ranked barriers or facilitators as seen in the present study. For example, during interviews professionals mentioned lack of knowledge about treatment options only a few times, whereas it was ranked as an important barrier for SDM. On the other hand lack of time during a consultation was mentioned often during interviews, and is also the most mentioned barrier for SDM in other studies [10]. In the present study, time during a consultation only took a 33th place, and did not occur in any of the professionals top 5. This emphasizes the importance of the ranking of barriers and facilitators after a qualitative study.

As professionals and patients mentioned different factors during (focus group) interviews, they therefore ranked different factors in the current study so that it is not possible to make an explicit comparison. However, many factors are related to each other. In view of the ranking of barriers and facilitators, there seems to be a need for more knowledge and information about sciatica and SDM, and skills to apply SDM. Therefore, healthcare professional training in knowledge regarding treatment options and SDM may improve SDM [22] and should be part of the implementation strategy. Another intervention may be the implementation of the existing decision aid for SDM in sciatica patients to facilitate information provision and SDM [22]. Furthermore, professionals working in primary or hospital care assigned a different importance to factors that may influence SDM, so that a multifaceted intervention is needed to integrate SDM in the complex multidisciplinary organization of sciatica care. For example who is responsible for which part of the information provision or guidance in which step of the care trajectory? Clear criteria are thus needed not only for (timing of) referral (especially important in primary care), but also regarding which part of the information on treatment is given by whom in which part of the care trajectory. The first mentioned intervention, training in knowledge and SDM will act on different factors. For example, professionals mentioned knowledge on treatment options, which is needed to provide information about both treatment options and potential harms and benefits to patients (which patients considered important). This training also gears at other important factors, such as skills to apply SDM, the importance for quick recovery of patient, and patients' willingness to decide. For example when professionals use SDM in their consultations the patients will tell them whether they want to recover quickly or not, and to determine patients willingness to decide is part of the SDM process. The second mentioned intervention, the use of a decision aid, may improve the interpretation of information by patients and the ability of patients to make the decision about treatment. Additionally, research has shown that patients are more likely to favor conservative treatments over surgery after patients' decision aid (DA) exposure [23], [24], which may lead to the reduction of preference-sensitive surgeries.

A strength of this study is the use of Maximum Difference scaling. MaxDiff is a relatively new method in health care research and was introduced by McIntosh and Louviere in 2002 [25]. As mentioned before, MaxDiff is scale free, and therefore prevents scale-use bias [18]. Furthermore, it is easy for respondents to complete, and results in ratio-scaled scores of importance [16], [26]. Factors with the highest importance score on average are not always the most important barriers or facilitators for all participants. A factor with a lower importance score can be considered as an important barrier by a smaller group of people. Therefore, it is important to take both the importance score and percentage of the total barriers or facilitators into consideration. Furthermore, we see that some factors are classified as both facilitator and barrier. This may reflect a difference in experience, where it was mainly a facilitator for some participants and a barrier for others, as they were asked to indicate this for their current situation. Another interpretation may be that it was difficult to classify a factor as a facilitator or barrier, especially for patients, given the neutral formulation of each factor. However, regardless of the interpretation, the ranking clearly shows which factors are more important than others for SDM to be implemented. A limitation of this study pertains to the recruitment of patients and professionals. This study is limited by its low response rate. Regarding the recruitment of patients, the procedure does not allow for a calculation of a response rate. It is possible that selection bias occurred, because patients who responded to the advisements may perceive the importance of barriers and facilitators differently than patients who did not respond. For the professionals, the response rate was only 30% of which 26% was included. Although this rate is relatively low, it is comparable to the response seen in another online survey (25% response rate) on the management of sciatica among physicians [27]. In addition, the response rate of online surveys is often lower compared to traditional surveys, due to server rejection, spam filters, automated forwarding or out-of-office replies [28]. Overall there is a decline in response rates over the past decades [29]. Especially GPs were extremely difficult to reach over email (15% response), possibly explained by that they see only a few sciatica patients per year. A similar lower response rate among GPs (18%) was also found in a previous study [27]. We recommend that future studies consider other approaches to reach respondents in order to improve the response to surveys, especially the response of the GPs. A more effective approach may be the presentation of a survey in power point slide format during a meeting of the target group with the response recorded upon entering a choice on a remote controlled device, as Raja et al. [30] (response 96%). Furthermore, it is possible that selection bias has occurred if professionals who do not use SDM in their consultation were less likely to complete the questionnaire, and experience other barriers and facilitators or rank them differently. We analyzed differences in groups of professionals who did and did not use SDM, and observed large overlap in their rankings even though there were some differences. Therefore, we think that the response rate does not bias the results of this study.

## Conclusions

This study showed the most important factors reported by patients and professionals for SDM implementation in sciatica care. Our study also demonstrates that the ranking of factors is an important step to determine which factors are the most important for which group of people, and thus on which factors an implementation strategy should be based. Several studies evaluated different interventions for an increase in the adoption of SDM among healthcare professionals, but there is a lack of evidence which type of intervention is the most effective [22]. Therefore, a multifaceted implementation strategy for SDM in sciatica care needs to be developed based on the most important factors as identified in this study. The effect of this strategy needs to be assessed to fill the gap between theories and clinical practice. This study focuses on SDM in sciatica care in the Netherlands, but the generated knowledge and understanding of the implementation process can also be used to implement SDM in other patients groups or other health care systems in which multiple disciplines are involved. Knowledge, information provision and a good relationship are the most important conditions for SDM perceived by both patients and professionals. These conditions are not restricted to one specific disease or health care system, because they are mostly professional or patient dependent and require healthcare professional training.
